# Microstructural and mechanical behaviours of Y-TZP prepared via slip-casting and fused deposition modelling (FDM)

**DOI:** 10.1016/j.heliyon.2023.e21705

**Published:** 2023-10-29

**Authors:** Constance L. Gnanasagaran, Karthikeyan Ramachandran, Nashrah Hani Jamadon, Vishaal Harikrishna Kumar, Andanastuti Muchtar, Ashwath Pazhani, Beenish Ayaz

**Affiliations:** a School of Engineering and the Environment, Kingston University, Roehampton Vale Campus, London, SW15 3DW, United Kingdom; b School of Mechanical Engineering, Coventry University, Coventry, CV1 2JH, United Kingdom; c Department of Mechanical and Manufacturing Engineering, Faculty of Engineering and Built Environment, University Kebangsaan Malaysia, 43600, Bangi, Selangor, Malaysia; d Department of Metallurgical Engineering, PSG College of Technology, Coimbatore, India

**Keywords:** Yttria-stabilized zirconia, Slip casting, Fused deposition modelling, Vickers hardness and indentation fracture toughness

## Abstract

This paper reports the microstructural characteristics and mechanical properties of yttria-stabilized zirconia prepared via fused deposition modelling and slip casting. X-Ray Diffraction peaks indicated that yttria-stabilized zirconia crystallized in tetragonal structure for both slip casted(SC) and fused deposition modelled(FDM) samples. Further, scanning electron microscopy of slip casted sample showcased closely packed structure with fine grains and an average grain size of ∼65 nm whilst fused deposition modelled samples showcased non-homogeneous pores with ∼20 nm grain size. Average relative density of slip casted samples was ∼99.4 % while that of fused deposition modelled sample exhibited ∼96.2 %. The Vickers Hardness of slip casted (∼15.26 ± 0.4 GPa) was ∼10 % higher than the fused deposition modelled samples (∼13.79 ± 0.3 GPa). Likewise, indentation fracture toughness of slip casted (5.78 ± 0.5 MPa m^1/2^) was 14 % higher than fused deposition modelled samples which could have been due to the change in grain size as well as porosity of the ceramics. Compressive strength of the fused deposition modelled samples was 32 % less than slip casted samples (∼510 ± 10 MPa) due to its non-homogenous pores which led to weakening van der Waals force of attraction.

## 1 Introduction

Currently, pursuit for lighter, compact, and high-performance ceramics are widely sought after in myriad of load bearing, heat exchanging and energy storing applications in aerospace, automotive and biomedical industries [[1], [2], [3]]. All these materials have one characteristic in common which is their predominantly high porosity [4,5]. Yttria-stabilized Zirconia (Y–TZP) nanoceramics attributed to the superior properties of zirconia such as very high wear resistance, commendable fracture toughness, chemical inertness, outstanding mechanical strength, low thermal conductivity, biocompatibility, and superior ionic conductivity has got worldwide interest in meeting the porosity criteria in recent years [6]. This material excels in machinery and mechanical applications including corrosion and insulating properties along with fatigue resistance and impact loading excellence [7]. In aerospace industry, porous Y-TZP has been particularly advantageous in reducing overall weight while avoiding the negative effects related to acoustics and vibration levels. Likewise, Y-TZP preserves its rigidity under extreme conditions and in biomedical applications, porous Y-TZP scaffolds are suitable for bone substitute materials for both macroscopic and microscopic reasons, [1,6,8,9]. In macroscopical view size, curvature, and shape of the pore enables the scaffold stiffness to match that of human bone and microscopically, a carefully optimized porosity can facilitate cell behaviour [8]. Hence, to achieve such properties which allow to reduce the overall weight of a structure while preserving its structural and behavioural integrity, careful consideration are required towards the design and manufacturing of the Y-TZP specific parts.

Traditionally, most industries utilize subtractive manufacturing techniques in which a material is successively removed from a solid block until the desired shape is acquired [9]. However, this method is not suitable for Y-TZP and other ceramics due to their brittle nature. Consequently, the next feasible route for ceramic manufacturing is to use conventional processing methods such as casting (slip/tape casting) [10]. Powder processing of fine Y-TZP materials enables the production of complex shaped parts with unique macro- and micro-structures which otherwise are impossible to achieve by using the subtractive manufacturing routes [11,12]. Slip casting is one of the commonly utilized colloidal processing techniques which is economically reasonable in terms of manufacturability and is capable of producing complex geometries [13]. Besides affordability, spontaneously formed soft agglomerates due to van der Waals attractive forces between the powder particles are broken down into individual particles and dispersed by promoting interparticle repulsion through slip-casting colloidal technique [14,15]. This leads to porosity initiation on the green body upon consolidation resulting from hard agglomerates left in the ceramic slurry [16]. Thus, a second consolidation technique such as cold isostatic pressing (CIP) is proposed to reduce particle agglomeration which supports enhancing the density [6,17]. However, even with the help of secondary consolidation, control over the density/porosity percentage for final ceramic component is challenging when processed through the conventional manufacturing routes.

Nevertheless, comparing to conventional manufacturing routes, Additive Manufacturing (AM) techniques like Fused Deposition Modelling (FDM) are found to be better in manufacturing ceramics with complex structures using various topology optimized porous shapes that are in demand across various industries [18]. For instance, not all AM in biomedical applications is found to be suitable for developing medical devices. Choosing an AM route over slip casting for fabrication in biomedical applications requires multiple success factors which could be achieved by optimizing the AM process parameters [19]. FDM offers and facilitates in achieving better micro-architectural features internally and in the bulk, which is more favorable in fabrication of biomedical components using Y-TZP [20]. FDM also allows important features like controlled porosity, shape/geometry of the porosity and lattice structure which significantly control the load bearing characteristics and damping mechanisms in additively manufactured parts [21].

Owing to the development in the manufacturing sector, the processing of ceramic based materials towards engineering sector has reached various peaks including use of additive manufacturing approaches. This research intends to compare and investigate the effect of fabrication techniques i.e., slip casting (SC) and fused deposition modelling (FDM) on the strength and microstructural integrity of Y-TZP to understand its utilization in load bearing applications.

## 2 Materials and methods

### 2.1 Materials

In this study, commercially available 3 mol.% Y-TZP nano-powder (US Research Nanomaterials Inc., Houston, TX, USA) with average particle size between 20 and 50 nm were obtained with purity of 99.95 %. For 3D printing through fused deposition modelling approach, zirconia filament with a chemical composition of 90 wt% zirconium oxide and 10 wt% organic binder system was obtained from Zetamix, France.

## 3 Methods

### 3.1 Additive manufacturing method

**Fig. 1:**
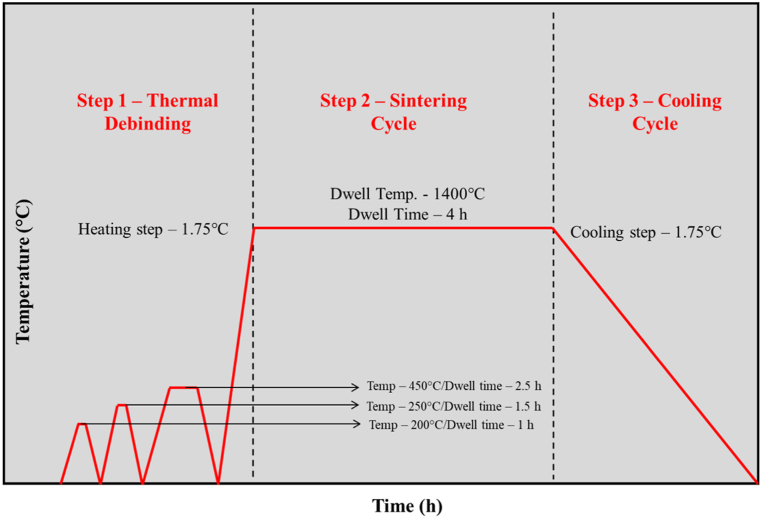
Temperature profile for thermal debinding and sintering zirconia through FDM approach.

Samples for FDM printing technique were developed through CAD modelling using SolidWorks 2020 in the dimensions of 12.1 mm in diameter and 6.1 mm in height. The design was developed by considering shrinkage factors obtained from the manufacturer. The designed model was exported to a slicing software (Ideamaker, Raise 3D, UK) where parameters like density and infill patterns were set. Towards this research, fully dense structures were printed with no infill patterns using (Raise 3D, Kingston University, London). The average print time for each sample was 6 min 22 s. The printed samples were then chemically debinded in acetone for 5 h at 40 °C using an ultrasonicator (Zetasinter, France) and rested overnight in room temperature. Further, thermal debinding was carried out in varying temperature (Tubular Furnace, Zetasinter, France) and dwell time and sintered at 1400 °C with heating and cooling rate of 1.75 °C/min and dwell time of 4 h as shown in Fig. 1.

### 3.2 Slip casting method

An aqueous suspension of nanocrystalline Y-TZP was prepared in accordance with the optimal polyethyleneimine (PEI) amount and pH range by mixing Y-TZP powder at the loading of 30 wt% with distilled water. The solution contained 0.4 wt% of 0.005 M PEI as dispersant and pH of distilled water was adjusted with the help of HNO_3_ to pH2 by optimization based on previous studies [6,17]. Using a magnetic stirrer, mixture was stirred for 30 min followed by ultra-sonification for 15 min. Then, suspension was stirred for another 15 min and further sedimented for 24 h. The sedimented solution was siphoned from vessels and large agglomerates were discarded. The attained supernatant was slip cast in a Teflon mould and further plaster of Paris block was utilized to absorb further moisture and dried in the drying cabinet for 48 h. The Y-TZP green bodies were then removed from the moulds after 2 days and extensive care was taken during the extraction, considering the fragility of the green bodies. The SC Y-TZP green bodies were then subjected to cold isostatic pressing (CIP) where the samples were placed in a vacuum glove for protection against oil contamination and then subjected to CIP (Riken Seiki, Japan) for 1 min at 200 MPa. High temperatures with long soaking times promote atomic diffusion and the densification of the final product [8]. These were sintered in a high temperature furnace at 1450 °C through a two-step process. The samples were sintered from room temperature to 600 °C with heating rate of 3 °C/min with dwell time of 45 min and further increased to desired temperature (1450 °C) with similar pattern with 4 h holding time and cooling rate of 5 °C/min.

### 3.3 Characterization and testing techniques

**Fig. 2:**
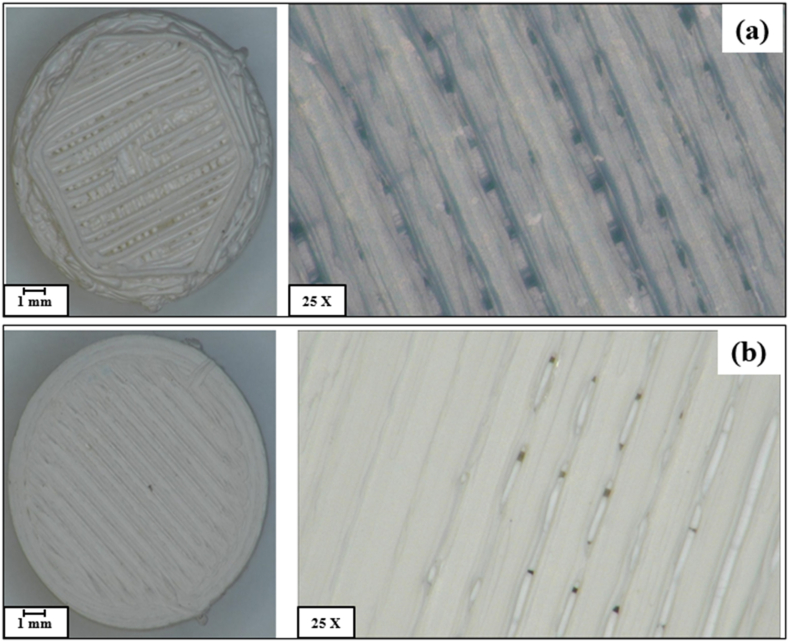
Fused deposition modelled Y-TZP samples (a) unsintered and (b) sintered.

The SC and FDM samples were characterized using X-Ray diffraction (Smart lab-RIGAKU, Japan) with varying 2θ values between 0° and 90° and current of 100 mA with wavelength of 1.54056 Å and step scan of 0.01 s and an operational target voltage of 30 kV. Scanning electron microscopy (Zeiss Evo) fitted with elemental distribution was utilized to understand the morphology and microstructure. The samples were coated with carbon nanoparticles using sputter coatings for characterization. The hardness of the Y-TZP prepared via SC and FDM were determined via Vickers indentation technique where the samples were subjected to a load on 10 kgF to through a diamond indenter for a period of 10 s. The attained diagonal impressions were measured through scanning electron microscopy and hardness values were determined. The Indentation fracture toughness of the sintered ceramics were also calculated by measuring the crack length with reference to the Evans–Charles equations [[22], [23], [24], [25]]. Compressive strength was measured through a universal testing machine (Zwick/Roell Z050) with maximum applicable load of 50 kN available at Kingston University, London. A steady force of 10 N was applied for 2 min on the samples and the stress-stain curves were devised and evaluated. Previous studies have provided optical microscopy results for SC samples [6,17] while Fig. 2(a) and (b) display images of both unsintered and sintered FDM samples respectively.

## 4 Results and discussion

### 4.1 Characterization & morphological behaviour

**Fig. 3:**
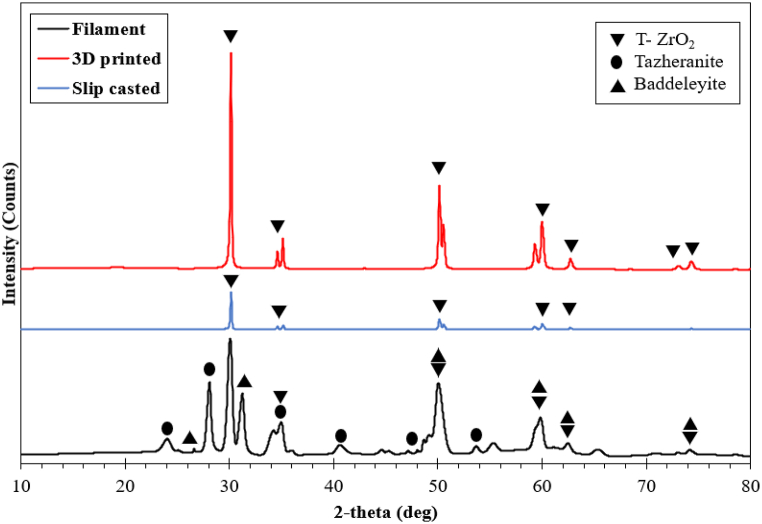
XRD spectrum of 3D zirconia filament and fabricated samples.

X-ray diffraction of the slip cast and FDM samples along with unprocessed zirconia filaments are represented in Fig. 3. The phases attained from zirconia filament indicated various forms of zirconium oxide i.e., tetragonal (JCPDS No. 01-075-9646), monoclinic structured baddeleyite (JCPDS No. 04- 005–4252) and tazheranite (JCPDS No. 04-011-9021). On the other hand, FDM samples represented only peaks of tetragonal phase of zirconium oxide throughout the surfaces which could have been due to the phase transformation of zirconium oxide during sintering process [26]. However, there were some noise peaks in some regions indicating the impurity in the samples. Similarly, SC samples also indicated presence of only tetragonal peaks on the surfaces without any significant noise [11,13]. This could be due to the utilization of 99.95 % pure Y- TZP powder as the primary material for slip casting as opposed to the filaments used for AM which contained some organic binders as reported in materials composition. The monoclinic phase is stable and has inferior strength at room temperature up to 1170 °C while the tetragonal phase is reported to be stable between 1170 °C and 2370 °C which has superior mechanical properties [12].

**Fig. 4:**
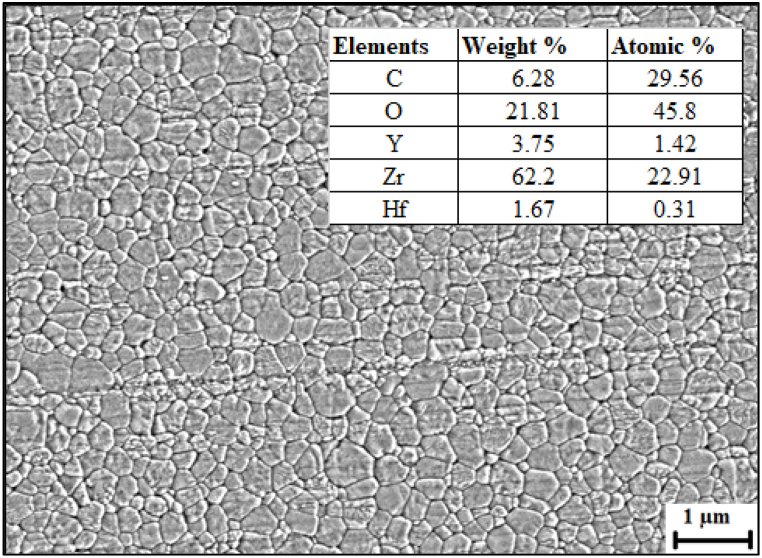
SEM microstructure and spot EDS of SC sample.

**Fig. 5:**
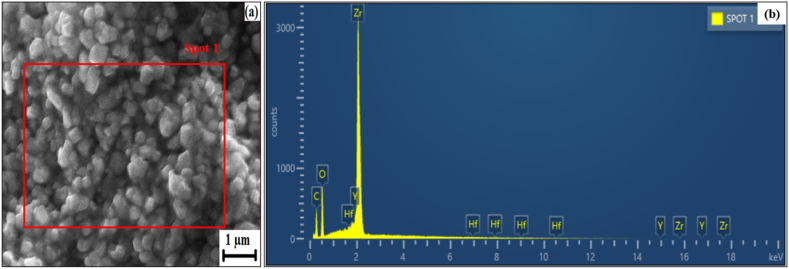
Microstructure of as-received zirconia filament and its corresponding EDS.

**Fig. 6:**
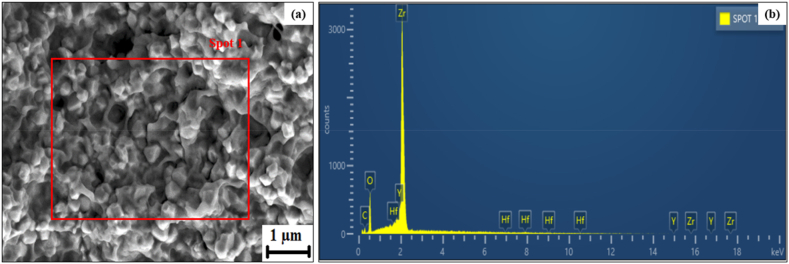
Microstructure of sintered zirconia and its corresponding EDS.

The microstructural features of the SC, FDM filament and fabricated samples are reported in Fig. 4, Fig. 5 and Fig. 6. From Fig. 4, it could be observed that SC samples were closely packed with a highly homogenous microstructure. The microstructure of SC samples exhibited very minimum to no pores which could have been due to the colloidal route and double consolidation process undertaken which reduces the number of internal pores [24]. The use of colloidal route resulted in homogenous distribution of the Y-TZP particles which was supported through EDS, and line intercept technique was utilized to measure the grain size. The SEM (Fig. 4) indicated a finer grain structure with average grain size of ∼65 nm. This could be due to the differences in the particle rearrangement and consolidation mechanisms between filament deposition and slip casting processes [27]. In slip casting, particles in a ceramic slurry settle and compact as liquid drains, allowing for close particle rearrangement and dense microstructure formation [6,12]. On the other hand, microstructure of 3D printed sample shown in Fig. 6 reveals the presence of numerous pores with nominal clusters. This contrasts with slip casting, where filament deposition arranges pre-formed filaments, leading to a less dense structure with a larger average particle size [18]. However, presence of pores in the 3D printed sample can be advantageous for specific applications such as tissue engineering, dental and bone implants [28]. Animal bones naturally contain complex and vital porosity parameters that are crucial for cell regeneration [29,30]. Thus, the non-homogeneous pore distribution achieved through filament deposition can mimic the pore distribution found in animal bodies [31].

**Fig. 7:**
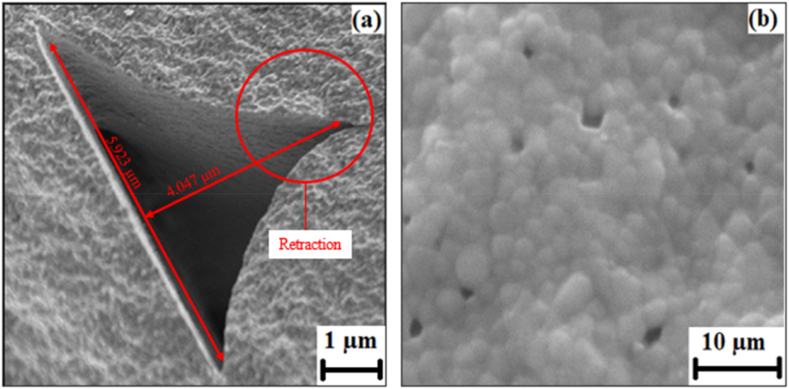
(a) Retraction from pores on the surface of FDM sample and (b) cross sectional area of FDM samples.

In Fig. 5(a), microstructure of the as-received zirconia filament is depicted, showing limited pores and a relatively homogeneous distribution of zirconia throughout the surface. In contrast, sintered zirconia, as evidenced in Fig. 6(a), exhibits a significant presence of porosity and a less homogeneous distribution. This proves that the density and porosity of a printed ceramic body are not solely dependent on the characteristics of the ceramic filament [32]. The printing process itself, including factors like filament bonding, consolidation, and the presence of trapped air or gas, significantly influences the final density and porosity. Achieving desirable density and minimizing porosity in FDM requires meticulous optimization of process parameters, including temperature, deposition speed, layer thickness, and post-processing treatments like sintering [33,34]. Also worth noting is the revelation of small traces of Hafnium (Hf), Yttrium (Y), and carbon by Energy Dispersive Spectroscopy (EDS) in both the as-received zirconia filament as can be seen in Fig. 5(b) and sintered zirconia illustrated in Fig. 6(b). Carbon is used for sputter coatings and is also part of the organic binder. The presence of Hf may be considered an impurity since Zr and Hf are often found together in mineral ore and obtaining a pure form is challenging even with advanced extraction and purification techniques [22,35]. The presence of pores on FDM samples could also be attributed to the manufacturing technique employed where the movement of the print head from one location to another at a designated retraction speed. This movement leave inhomogeneities which lead to pores and the retraction was conspicuous on the edges as illustrated in Fig. 7(a) [32]. These pores were observed to be approximately ∼5.9 μm in length, as measured by SEM. The morphological cross section of the FDM sample is illustrated in Fig. 7(b)shows non homogenous pores on Y-TZP with no visible cracks on the surfaces with limited to almost zero cracks propagating from the pores, showcasing the stability of the 3D printed YS-Zirconia. This could be due to grain growth and stress relief on the sample at high sintering temperature [36]. The particle size of the FDM samples was determined using the linear intercept technique, showing a range of particle sizes from a minimum of 13 nm to a maximum of 28 nm.

The densification behaviour of SC and FDM samples are reported in Table 1 upon completion of sintering, the average relative density of SC samples was reported to be ∼99.4 % which is close to the theoretical density. These attained results were similar to a previous report on the relative density of the Y-TZP [37]. This could be attributed to the CIP technique which enhances the compact bonding of Y-TZP particles. The enhancement in the relative density might also be due to colloidal processing which reduces the gaps between the particles, which upon moulding results in a dense structure. Also, sintering temperature was adequate to eliminate porosity on the microstructure along with structure densification. On the other hand, FDM sample had an average relative density of ∼96.2 %. The decrease in density could be due to the micro-porous gaps on the surfaces and volume of the sintered bodies as indicated in Fig. 7(b). Even though sintering temperature on both fabrication conditions i.e., slip casting (1450 °C) and additive manufacturing (1400 °C) were close, the AM approach of printing by FDM leaves inhomogeneities, which mature to form pores due to thermal contraction of the fused material upon solidification. This generation of pores decreases the apparent density of the final product. The correlation between the drop in density with increase in number of pores has been reported by various researchers [[37], [38], [39], [40]]. Also, pores could also develop due to agglomeration of Y-TZP which may result in continuous open pore networks which inhibits Joule heating leading to extensive agglomeration as well as density reduction [25,41]. The reduction in density due to an increased number of pores or agglomeration is a significant concern in additive manufacturing processes, as it can impact the mechanical properties, structural integrity, and overall performance of the printed component as can be understood from the following discussion [32]. Therefore, optimizing process parameters and employing techniques to minimize pore formation and agglomeration are essential to achieve higher density and improved material properties in additive manufacturing.

**Table 1:** Relative density of Y-TZP fabricated via slip casting and additive manufacturing.

Fabrication Technique	Average Relative Density (%)
Slip Casting	∼99.4 %
Additive Manufacturing	∼96.2 %

## 5 Mechanical properties

### 5.1 Compressive strength and Young's modulus

**Fig. 8:**
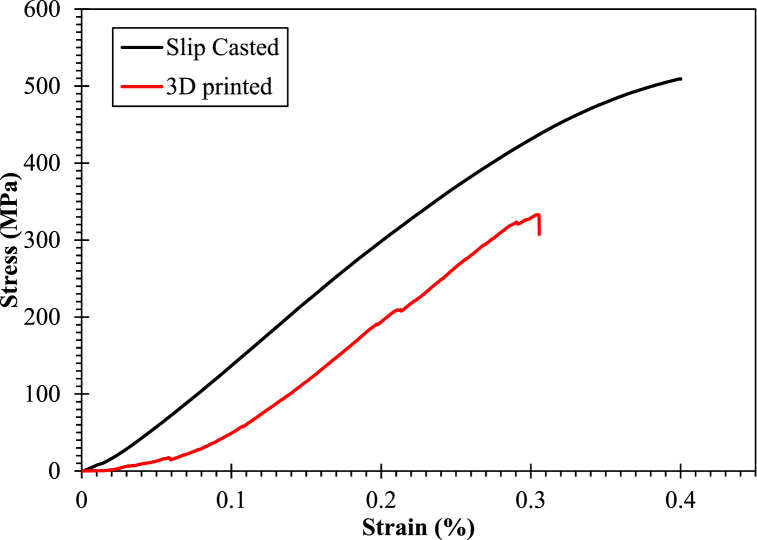
Average stress-strain compressive strength curve for fabricated Y-TZP composites via slip casting and 3D printed techniques.

The compressive strength of Y-TZP samples prepared via both fabrication techniques were measured and represented as a stress-strain curve in Fig. 8. From Fig. 8, it could be observed that SC samples had a maximum stress of ∼510 ± 10 MPa whereas 3D printed sample had a maximum strength of ∼332 ± 10 MPa which was about ∼32 % decrease than SC samples. The remarkable compressive strength of the SC Y-TZP could be due to the highly packed structure and elevated sintering temperature. When the sintering temperature is increased, the remaining pores on the surface of the ceramic close (i.e., transformation of open pores to closed pores occurs due to the heat which enhance the van der Waals force of attraction) and grains start to go through necking [24,37,42]. The SC samples showcased typical brittle failure mode of the ceramics with no fracture lines visible on the surface of the sample. This is a typical scenario for highly dense structures where the absence of porosity directly effects the toughness of the material. On the other hand, FDM samples indicated a drop in compressive strength which could be due to higher number of pores resulted from the fabrication technique [42,43]. The presence of numerous pores on the surfaces of the ceramics induced a pseudo-brittle behaviour as illustrated in Figure 8 which led to the drop in the compressive strength of the FDM samples [44]. Further, the overall compressive strength is also relative to the mineral content of the samples. Hansson et al. reported that the compressive strength was increasing linearly with amounts of bone mineral content. Hence, with a more architecturally relevant structures obtained via FDM, bone mineralization can be optimized to achieve desired compressive strength [45]. The calculated Young's modulus of SC samples were about ∼16 GPa and ∼14 GPa for the FDM. These attained results were quite promising, as both values were in the acceptable range for real animal dentin, particularly FDM showcasing a promising future to be used to construct TE dentins [46].

### 5.2 Hardness & fracture toughness

**Fig. 9:**
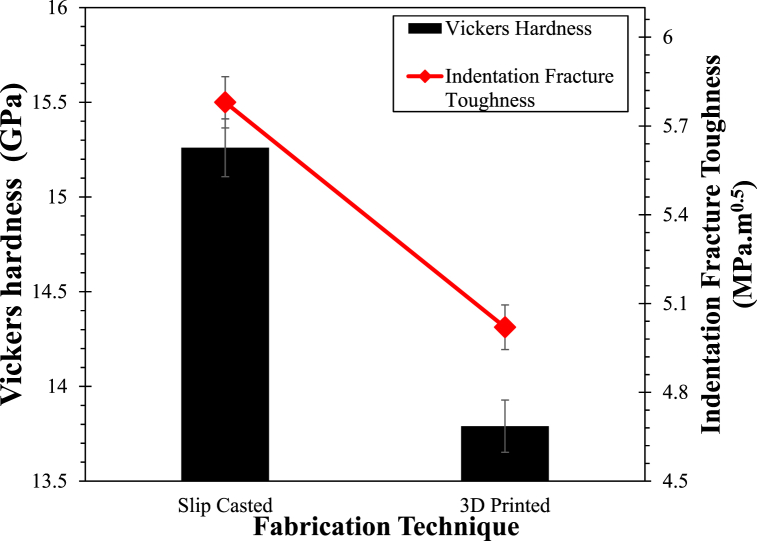
Vickers hardness and indentation fracture toughness of T-YPZ fabricated via SC and FDM samples.

Vickers hardness of the Y-TZP was carried out and the indentation diagonals were measured through scanning electron microscopy. The average Vickers hardness of the fabricated Y-TZP is reported in Figure 9. The average hardness of the SC sample was about 15.26 ± 0.4 GPa which was higher than some of the previous reported research [16,25]. This enhanced hardness could be due to colloidal route in which slip casting was processed, which led to the homogeneous suspension of the Y-TZP resulting in better interaction between the atoms during sintering process. Further, increase in the hardness could be attributed towards phase changes during sintering which could result in the modification of grain size [47]. The line intercept technique was used to understand the grain size of the Y-TZP pre and post-sintering, and it was determined that the average grain size of the ceramic prior to sintering was between ∼16 nm (min.) - ∼24 nm (max.) which further enhanced to ∼49 nm (min.) – ∼63 nm (max.) post sintering. The increase in grain size falls under the reported range where inverse Hall-Petch relation is believed to operate. That could support the hardness enhancement and was also validated through SEM technique. The average hardness of the FDM sample was 13.79 ± 0.3 GPa which was about ∼10 % lower than the SC samples. This drop in the hardness could be due to the fabrication technique which resulted in high porosity throughout the surfaces of the FDM samples.

**Fig. 10:**
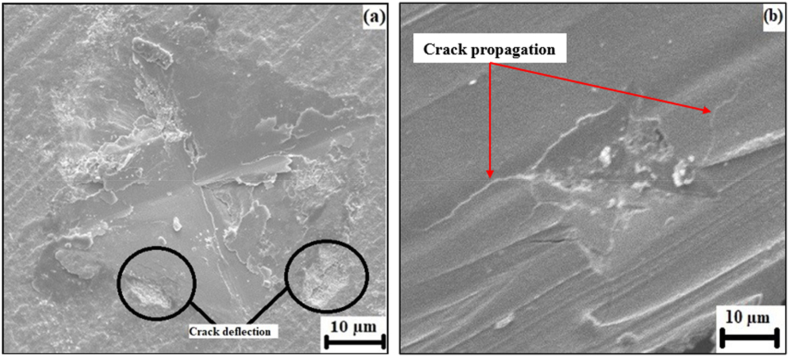
Vickers hardness indentation on ceramics fabricated via (a) SC and (b) FDM samples.

Indentation fracture toughness of Y-TZP fabricated via both routes was calculated through Evans – Charles equation and is reported in Fig. 9 along with Vickers hardness. The SC fabricated samples had an indentation fracture toughness of 5.78 ± 0.5 MPa m^0.5^ which was within the range as in previously reported research [7,25]. Fig. 10(a) reports the fractographic SEM of cracks formed after indentation with a diamond intender on the surface of ceramics showcasing with limited crack propagation and showcasing crack deflection mechanism on the SC samples. The crack deflection mechanism was responsible for higher fracture toughness of Y-TZP as it supported the energy-dissipation at the interfaces on the surfaces [40]. The fracture toughness of additively manufactured Y-TZP was about 5.02 ± 0.3 MPa m^0.5^ which was about ∼14 % less than the SC samples. This could be due to the fabrication technique which initiated more pores on the surfaces of Y-TZP [18]. Pores in the printed material, formed during the retraction of the print head and solidification process, tend to align linearly and act as precursor sites for crack initiation and propagation [48,49]. The presence of these aligned pores reduces energy dissipation on the material surfaces, leading to a decrease in crack deflection and facilitating easier and faster crack propagation, particularly from the corners of indentation diagonals [50]. Observations from the SEM image depicted in Fig. 10(b) reveal that crack propagation is more pronounced in the direction perpendicular to the print direction. This unique behaviour, to the best of our knowledge, has not been reported previously. The higher crack propagation in this direction could be attributed to axial locked-in stresses acting perpendicular to the applied load. These stresses may induce preferential crack propagation on the surfaces due to the inherently brittle behaviour of ceramics [3]. The alignment of pores and the resulting crack propagation patterns perpendicular to the print direction highlight the importance of considering the anisotropic mechanical behaviour of printed ceramics [7,10]. Understanding and characterizing such phenomena are crucial for optimizing material properties, designing reliable components, and developing strategies to mitigate crack propagation in ceramic-based additive manufacturing processes.

## 6 Conclusion

This research study investigated two different fabrication approaches, Fused Deposition Modelling (FDM) and slip casting (SC) for Y-TZP ceramics in load-bearing applications. X-ray diffraction analysis confirmed the presence of a tetragonal structure in both SC and FDM samples, indicating their suitability for load-bearing purposes with higher strength compared to monoclinic phases. Microstructural analysis revealed that SC samples exhibited a closely packed structure with a homogeneous distribution of coarse grains, averaging ∼65 nm in size. The increase in grain size could be attributed to grain growth during sintering of the compactly packed powder through cold isostatic pressing. On the other hand, FDM samples demonstrated numerous non-homogeneous pores, averaging ∼20 nm in size, resulting in a relative density reduction of approximately 5 % compared to SC samples. However, presence of such non-homogeneous pores is preferred in tissue engineering applications, as it mimics the inherent porosity found in natural hard tissues. Mechanical property analysis indicated that SC samples outperformed FDM samples by exhibiting higher hardness, fracture toughness, and compressive strength by 10 %, 14 %, and 32 % respectively. These superior mechanical properties of SC samples can be attributed to their higher density and strong van der Waals forces of attraction. On the other hand, FDM enables the fabrication of more biologically relevant structures with precise control over the manufacturing process and final product.

These findings highlight the trade-offs between the two fabrication approaches. SC provides samples with superior mechanical properties, making it well-suited for load-bearing applications that require high strength and density. In contrast, FDM offers advantages in producing structures with biomimetic porosity, which is advantageous for tissue engineering applications. Future research efforts could focus on optimizing FDM to enhance density and mechanical properties, as well as exploring hybrid approaches that combine the strengths of both SC and FDM. This would enable the development of advanced ceramic structures with tailored properties for specific load-bearing and tissue engineering applications. In conclusion, this research contributes valuable insights into the utilization of FDM and SC for Y-TZP ceramics in load-bearing applications. The results demonstrate the potential for both approaches to be utilized in different engineering and biomedical fields, with SC offering superior mechanical properties and FDM enabling the fabrication of biologically relevant structures with precise control.

## Funding

This research was funded by First Kickstarter Grant (P2186-133) by 10.13039/100010049Kingston University London.

## Code availability

No Codes were utilized as part of this research.

## Ethical approval

Not Applicable.

## Data availability

The data supporting the findings of this study are available upon request. Please contact Constance L Gnanasagaran at constance.g@kingston.ac.uk or Karthikeyan Ramachandran at K1825123@kingston.ac.uk to request access to the data.

## CRediT authorship contribution statement

**Constance L. Gnanasagaran:** Project administration, Methodology, Investigation, Funding acquisition, Formal analysis, Data curation, Writing - original draft. **Karthikeyan Ramachandran:** Visualization, Validation, Resources, Project administration, Methodology, Conceptualization, Funding acquisition, Software, Writing - original draft, Writing - review & editing. **Nashrah Hani Jamadon:** Supervision, Resources, Methodology, Formal analysis, Conceptualization. **Vishaal Harikrishna Kumar:** Validation, Software, Resources, Project administration. **Andanastuti Muchtar:** Validation, Supervision, Software, Resources, Project administration. **Ashwath Pazhani:** Writing - review & editing. **Beenish Ayaz:** Writing - review & editing.

## Declaration of competing interest

The authors declare that they have no known competing financial interests or personal relationships that could have appeared to influence the work reported in this paper.

## References

[1] Göransson P. (2008). Tailored acoustic and vibrational damping in porous solids – engineering performance in aerospace applications. Aero. Sci. Technol..

[2] Michailidis N., Tsouknidas A., Lefebvre L.-P., Hipke T., Kanetake N. (2014). Production, characterization, and applications of porous materials. Adv. Mater. Sci. Eng..

[3] Gnanasagaran C.L., Ramachandran K., Ramesh S., Ubenthiran S., Jamadon N.H. (2023). Effect of co-doping manganese oxide and titania on sintering behaviour and mechanical properties of alumina. Ceram. Int..

[4] Altenback H. (2011). Mechanics of advanced materials for lightweight. J. Mech. Eng. Sci..

[5] Scheithauer U., Kerber F., Fussel A., Holtzhausen S., Beckert W., Schwarzer E., Weingarten, Michaelis A. (2019). Alternative process routes to manufacture porous ceramics—opportunities and challenges. Materials.

[6] Chin C.H., Muchtar A., Azhari C.H., Razali M., Aboras M. (2018). Influences of the processing method and sintering temperature on the translucency of polycrystalline yttria-stabilized tetragonal zirconia for dental applications. Ceram. Int..

[7] Coric D., Renjo M.M., Curkovic L. (2017). Vickers indentation fracture toughness of Y- TZP dental ceramics. Int. J. Refract. Metals Hard Mater..

[8] Lu Y., Cheng L., Yang Z., Li J., Zhu H. (2020). Relationship between the morphological, mechanical and permeability properties of porous bone scaffolds and the underlying microstructure. PLoS One.

[9] Md-Aracil, Perez J., Morenilla A., Hm-Mora (2018). 3D printing of functional anatomical insoles. Comput. Ind..

[10] Lakhdar Y., Tuck C., Binner J., Terry A., Goodridge R. (2021). Additive manufacturing of advanced ceramic materials. Prog. Mater. Sci..

[11] Aboras M., Muchtar A., Azhari C.H., Yahaya N., Mah J.C.W. (2019). Enhancement of the microstructural and mechanical properties of dental zirconia through combined optimized colloidal processing and cold isostatic pressing. Ceram. Int..

[12] Franks G., Tallon C., Studart A.R., Sesso M., Leo S. (2017). Colloidal processing: enabling complex shaped ceramics with unique multiscale structures. Journal of Americal Ceramic Society.

[13] Hao C.C., Muchtar A., Azhari C.H., Razali M., Aboras M. (2016). Fabrication of Y-TZP for dental crowns applications by combining slip casting and cold isostatic pressing. Malaysian Journal of Analytical Sciences.

[14] Tallon C., Limacher M., Franks G. (2010). Effect of particle size on the shaping of ceramics by slip casting. J. Eur. Ceram. Soc..

[15] Rao R.R., N R.H., S K.T. (2001). pH controlled dispersion and slip casting of Si3N4 in aqueous media. Bull. Mater. Sci..

[16] Garmendia N., Santacruz I., Moreno R., Obieta I. (2009). Slip casting of nanozirconia/MWCNT composites using a heterocoagulation process. J. Eur. Ceram. Soc..

[17] Chin C.H., Muchtar A., Azhari C.H., Razali M., Aboras M. (2015). Optimization of pH and dispersant amount of Y-TZP suspension for colloidal stability. Ceram. Int..

[18] Mirzaali M., Moosabeiki V., Rajaai S., Zhou J., A, Zadpoor (2022). Additive manufacturing of biomaterials—design principles and their implementation. Materials.

[19] Wang X., Xu S., Zhou S., Xu W., Leary M., Choong P., Qian M., Brandt M., Y, Xie (2016). Topological design and additive manufacturing of porous metals for bone scaffolds and orthopaedic implants: a review. Biomaterials.

[20] Arnesano A., Padmanabhan S., Notarangelo A., Montagna F., A, Licciulli (2020). Fused deposition modeling shaping of glass infiltrated alumina for dental restoration. Ceram. Int..

[21] Wang J., Xie H., Weng Z., Senthil T., L, Wu (2016). A novel approach to improve mechanical properties of parts fabricated by fused deposition modeling. Mater. Des..

[22] Mudiyanselage Y.C.J., Ramachandran K., Jayaseelan D.D. (2022). Fabrication and characterisation of ZrSi2 ceramics via reactive hot-pressing. Adv. Appl. Ceram..

[23] Ramachandran K., Jayakody Y.C., Jayaseelan D.D. (2023). Oxidation behaviour and its effect on fracture toughness of Niobium metal. Int. J. Refract. Metals Hard Mater..

[24] Ramachandran K., Subramani R., Arunkumar T., Boopalan V. (2021). Mechanical and thermal properties of spark plasma sintered Alumina-MWCNTs nanocomposites prepared via improvised colloidal route. Mater. Chem. Phys..

[25] Arunkumar T., Anand G., Subbiah R., Karthikeyan R. (2021). Effect of multiwalled carbon nanotubes on improvement of fracture toughness of spark-plasma-sintered yttria- stabilized zirconia nanocomposites. J. Mater. Eng. Perform..

[26] Bocanegra-Bernal M., Torre S.D.d. l. (2002). Phase transitions in zirconium dioxide and related materials for high performance engineering ceramics. J. Mater. Sci..

[27] Kim M.-J., Ahn J.-S., Kim J.-H., Kim H.-Y., Kim W.-C. (2013). Effects of the sintering conditions of dental zirconia ceramics on the grain size and translucency. The Journal of Advanced Prosthodontics.

[28] Collins M.N., Ren G., Young K., Pina S., Reis R.L., Oliveira J.M. (2021). Scaffold fabrication technologies and structure/function properties in bone tissue engineering. Adv. Funct. Mater..

[29] Gao C., Peng S., Feng P., Shua C. (2017). Bone biomaterials and interactions with stem cells. Bone Research.

[30] Mohammadi H., Sepantafar M., Muhamad N., Sulong A.B. (2021). How does scaffold porosity conduct bone tissue regeneration?. Adv. Eng. Mater..

[31] Masciandaro S., Torrell M., Leone P., Tarancon A. (2019). Three-dimensional printed yttria-stabilized zirconia self-supported electrolytes for solid oxide fuel cell applications. J. Eur. Ceram. Soc..

[32] Maurath J., Willenbacher N. (2017). 3D printing of open-porous cellular ceramics with high specific strength. J. Eur. Ceram. Soc..

[33] Lakhdar Y., Tuck C., Binner J., Terry A., Goodridge R. (2021). Additive manufacturing of advanced ceramic materials. Prog. Mater. Sci..

[34] Chen Z., Li Z., Li J., Liu C., Lao C., Fu Y., Liu C., Li Y., Wang P., He Y. (2019). 3D printing of ceramics: a review. J. Eur. Ceram. Soc..

[35] Srichumpong T., Pintasiri S., Heness G., Leonelli C., Meechoowas E., Thogpun N., Teanchai C. (2021). The influence of yttria-stabilised zirconia and cerium oxide on the microstructural morphology and properties of a mica glass-ceramic for restorative dental materials. Journal of Asian Ceramic Societies.

[36] Zhang F., Spies B.C., Willems E., Inokoshi M., Wesemann C., Cokic S.M., Hache B., Kohal R., Altmann B., Vleugels J., Meerbeek B.V., Rabel K. (2022). 3D printed zirconia dental implants with integrated directional surface pores combine mechanical strength with favorable osteoblast response. Acta Biomater..

[37] Amat N.F., Muchtar A., Ghazali M.J., Yahaya N. (2014). Suspension stability and sintering influence on yttria-stabilized zirconia fabricated by colloidal processing. Ceram. Int..

[38] Pradhan M., Kapur P., Pradip (2012). Effect of powder dispersion on sintering behaviour and mechanical properties of nanostructured 3YSZ ceramics. Ceram. Int..

[39] Mazaheri M., Simchi A., Golestani-Fard F. (2008). Densification and grain growth of nanocrystalline 3Y-TZP during two-step sintering. J. Eur. Ceram. Soc..

[40] Thirugnanasabandam A., Ramachandran K., R R.S., Mariadas A., Jayaraman J.T., Boodula R., Jagannathan M. (2020). Effect of MWCNTs on improvement of fracture toughness of spark plasma sintered SiC nano-composites. Curr. Anal. Chem..

[41] Hassan R., Nisar A., Ariharan S., Alam F., Kumar A., Balani K. (2017). Multi-functionality of carbon nanotubes reinforced 3 mol% yttria stabilized zirconia structural biocomposites. Mater. Sci. Eng., A.

[42] Zhou J., Wang C.-A. (2012). Porous yttria-stabilized zirconia ceramics fabricated by nonaqueous-based gelcasting process with PMMA microsphere as pore-forming agent. J. Am. Ceram. Soc..

[43] V D., A. T.-O. J. A. P. E. X. a. F. F.-A. Irene Buj-Corral (November 2021). Characterization of 3D printed yttria-stabilized zirconia parts for use in prostheses. Nanomaterials.

[44] Kumar S., Dhas B., Roy D. (2018). Emergence of pseudo-ductility in laminated ceramic composites. Compos. Struct..

[45] Hansson T., Roos B., Nachemson A. (1980). The bone mineral content and ultimate compressive strength of lumbar vertebrae. Spine.

[46] Zuccarini C., Ramachandran K., Russo S., Jayakody Y., Jayaseelan D. (2022). Mathematical modeling and simulation of porosity on thermomechanical properties of UHTCs under hypersonic conditions. International journal of Ceramics Engineering & Science.

[47] Hall S., Fischer T., Gruffel P., Carry C. (1995). Effect of grain boundary dopants and mean grain size on tribomechanical behaviour of highly purified α-alumina in the mild wear regime. Wear.

[48] Gnanasagaran C.L., Ramachandran K., Ramesh S., Ubenthiran S., Jamadon N.H. (2023). Effect of co-doping manganese oxide and titania on sintering behaviour and mechanical properties of alumina. Ceram. Int..

[49] Zocca A., Colombo P., Gomes C.M., Günster J. (2015). Additive manufacturing of ceramics: issues, potentialities, and opportunities. Journal of the Americal Ceramic Society.

[50] Ligon S.C., Liska R., Stampfl J., Gurr M., Mülhaupt R. (2017). Polymers for 3D printing and customized additive manufacturing. Chem. Rev..

